# Supplementary material for: NRMLncR, a myocyte-enriched long non-coding RNA, enhances myogenesis in mouse
Source: bioRxiv. 2026 Feb 14:2026.02.11.704964. Preprint. [Version 2] doi: 10.64898/2026.02.11.704964 (PMC12918952; doi:10.64898/2026.02.11.704964)
Supplement: Supplement 1 [file media-1.pdf]

Supplemental Material

Supplementary Table 1

List of primers used in this study

Application	Gene name	Primer sequence (5'-3')
qPCR	*18s*	F: AGTCCCTGCCCTTTGTACACA
		R: CGATCCGAGGGCCTCACTA
qPCR	*U6*	F: CTCGCTTCGGCAGCACA
		R: AACGCTTCACGAATTTGCGT
qPCR	*β-actin*	F: GGCTGTATTCCCCTCCATCG
		R: CCAGTTGGTAACAATGCCATGT
qPCR	*NRMLncR-v1/2*	F: CGGATAGCAGAGGCAGAGATGAG
		R: CAGCCCAGGAGGAGACTTCAT
qPCR	*NRMLncR-v1*	F: AAGATGGTGACCTTGCGTCCT
		R: GCATCAGGCGTGTGTCCAG
qPCR	*Pax7*	F: CGACTCTGGATTCGTCTCC
		R: GGCCTTGGCCAAGAGGG
qPCR	*MyoD*	F: GGCTACGACACCGCCTACTA
		R: CGACTCTGGTGGTGCATCTG
qPCR	*MyoG*	F: TGCCCAGTGAATGCAACTCC
		R: TTGGGCATGGTTTCGTCTGG
qPCR	*eMyhc*	F: AAAAGGCCATCACTGACGC
		R: CAGCTCTCTGATCCGTGTCTC
qPCR	*Myh8*	F: GGAGAGGATTGAGGCCCAAAA
		R: CACGGTCACTTTCCCTCCATC
qPCR	*Hey1*	F: TGAATCCAGATGACCAGCTACTGT
		R: TACTTTCAGACTCCGATCGCTTAC
qPCR	*Hey2*	F: AAGCGCCCTTGTGAGGAAAC
		R: GGTAGTTGTCGGTGAATTGGAC
qPCR	*Hes2*	F: ACAATTACCCTGGGCACGCTAC
		R: CCTGTAGCCTGGAGCATCTTCAAA
qPCR	*Heyl*	F: CAGATGCAAGCCCGGAAGAA
		R: ACCAGAGGCATGGAGCATCT
qPCR	*Follistatin*	F: GCCAGTGACAATGCCACATACG
		R: CTTCCTCCGTTTCTTCCGAGATG
qPCR	*Ap2m1*	F: GACATCGGGAGGAATGCTGTG
		R: TGGACCGCTTAACATGGAAGA
qPCR	*Cdc45*	F: GATTTCCGCAAGGAGTTCTACG
		R: TACTGGACGTGGTCACACTGA
qPCR	*Dvl3*	F: GTCACCTTGGCGGACTTTAAG
		R: AAGCAGGGTAGCTTGGCATTG
qPCR	*Eif2b5*	F: AGTTCTAGTGGCCGATAGCTT
		R: AGCAGCAAAAGACAAATGTTTCC
qPCR	*Etv5*	F: TCAGTCTGATAACTTGGTGCTTC
		R: GGCTTCCTATCGTAGGCACAA
qPCR	*Hira*	F: CCACCGTTCGGGGGATAAG
		R: GGCAACACATACCACATCACAG
qPCR	*Igf2bp2*	F: GTCCTACTCAAGTCCGGCTAC
		R: CATATTCAGCCAACAGCCCAT
qPCR	*Klhl6*	F: GCTTGGAAGGACCCTTAGCAC
		R: CGTCTGTCAAAGCATTTTCCTCT
qPCR	*Lamp3*	F: CAAGGACAGATCAACGACCTC

		R: GCCTGCTTCCATTTAGGACTTC
qPCR	*Map3k13*	F: CCCGACCTCATCTCCACAG
		R: TGGAAACAGGGATCATAGGGTT
qPCR	*Parl*	F: TACGGCCACAAAAGGAAGGAA
		R: TTCGCAGCTATGATGCCTGTC
qPCR	*Senp2*	F: GCTGGCTAAGGTTCTCGGC
		R: CTGGGATCTCATCAGTGTCCA
qPCR	*Sept5*	F: GAAAGGTTTCGACTTCACGCT
		R: CCGGTCCTTATACAGGTCGGT
qPCR	*Thpo*	F: GGCCATGCTTCTTGCAGTG
		R: AGTCGGCTGTGAAGGAGGT
qPCR	*Ccnd1*	F: GCGTACCCTGACACCAATCTC
		R: CTCCTCTTCGCACTTCTGCTC
qPCR	*β-arrestin1*	F: CCTGCATCAGCCAGATGAAG
		R: GGTGTTTCAGGATGGCCTTC
PCR	*NRMLncR*	F: ACCTTGAGTGATCTTCAGGGAGA
		R: GGCATCAGGCGTGTGTCCAG
5'UTR+ORF	*NRMLncR*	F: CGCTCTAGAAGATGGTAAATTCCCTTAGCA
		R: TTTGGATCCTCGATGGTTTGCAGACTGCAGC
ORF	*NRMLncR-v1*	F: CGCTCTAGAATGGAGGCCAGAGAAGATG
	NRMLncR-v1-mutant	F: CGCTCTAGATAGGAGGCCAGAGAAGATG
ORF	*NRMLncR-v2*	F: TATTCTAGAATGGTGGTGCGGATAGCAG
	NRMLncR-v2-mutant	F: TATTCTAGATAGGTGGTGCGGATAGCAG
ORF	*Hey1*	F: CGCTCTAGAATGAAGAGAGCTCACCCAGACT
		R: TTTGGATCCGAAAGCTCCGATCTCTGTCC
Promoter	*NRMLncR*	F: TTTACGCGTAGCTTTGAATCAGCAGGACTGTC
		R: GGGCTCGAGTAAGGGAATTTACCATCTAAGG
ChIP-PCR	E-box site 1/2	F: CAGCAAGACAGAGCATTCTTCAC
		R: CAAACGGTAAACGGCTCAAAAG
ChIP-PCR	E-box site 3	F: AGAACCCTGGATCTAGTAGCTGAG
		R: GGCTCTGAGTTGACCTCTTCATT
shRNA	NRMLncR-shRNA-1	F:CCGGGGAGGTTTAACTATACCAATGCCACACCCATTGGTATAGTTAAACCTCCTTTTTG
		R:AATTCAAAAAGGAGGTTTAACTATACCAATGGGTGTGGCATTGGTATAGTTAAACCTCC
shRNA	NRMLncR-shRNA-2	F:CGGGGAGGTTTAACTATACCAATGCCACACCCATTGGTATAGTTAAACCTCCTTTTTG
		R:TCGAGAAAAAGCAGTCTGCTTGTATTCAACACTCGAGTGTTGAATACAAGCAGACTGCT
In vitro transcription	*NRMLncR-v1*	F: TAATACGACTCACTATAGGGAGATGGTAAATTCCCTTAGCA
		R: GGGTATCAACTTTTACTCTCTCC
Adenoviral OE	*NRMLncR-v1*	F: TTTGCGGCCGCAGATGGTAAATTCCCTTAGCA
		R: GCGGATATCGGGTATCAACTTTTACTCTCTCC
